# Effects of mechanical forces on the formation of cutaneous wounds during skin expansion and emerging therapies for wound healing and scar prevention

**DOI:** 10.15537/smj.2023.44.1.20220556

**Published:** 2023-01

**Authors:** Jiménez Ibañez E. Oswaldo, Domenico Tripodi, Yousuf Al-Shaqsi, Oscar E. Flores Woods, Rodrigo Valero

**Affiliations:** *From the Plastic, Aesthetic and Reconstructive Surgery Mexico City (Oswaldo, Eduardo), from the Lucerna Medical Center, German Gedovius Tijiuana Mexico (Valero), Mexico, from the Department of Surgical Sciences (Tripodi), Sapienza University of Rome, Rome, Italy, and from the Division on Pediatric Urology (Al-Shaqsi), CHU Sainte- Justine, Montreal, Canada.*

**Keywords:** scar prevention, wund healing, keloid, cosmetic scar

## Abstract

**Objectives:**

To update a possible role of cosmeceutical topic treatment to obtain a better scar.

**Methods:**

This is a preliminary supportive study. A total of 14 patients who went to the General Hospital of Mexico City, Mexico, between May and December 2020, for breast reconstruction were included in the current study. The biopsies were carried out to the scar area of the previous I° and II° surgery. The patients were thus divided into 2 groups: those who used Cicolea cream® as a treatment supplement and those who used only petrolatum.

**Results:**

Collagen fibers arranged in a regular pattern in the group treated with Cicolea compared to dispersed collagen fibers in the group treated with pure petrolatum. Furthermore, the patients who presented hypertrophic or keloid scars secondary to mastectomy, developed after insertion of breast expanders an organized scarring process, with improvement of scar if treated with Cicolea.

**Conclusion:**

Based on our observations, it is possible to propose that the action of the polyphenols present in the different components of Cicolea® cream leads to a better evolution of the wound healing compared to the action of petrolatum composition.

**T**he skin is mechanically stressed by various external stimuli, but the effects of mechanical stress on human skin are not fully understood. It is believed to stimulate epidermal proliferation. Mechanical stress is naturally a consequence of the rapid expansion of abdominal skin during pregnancy, weight loss or gain, or somatic development in women. When the skin has limited ability to adapt to the stress, there is severe damage to the collagen fibers and the formation of stretch marks on the skin. The stretch marks are associated with a dormant phenotype of dermal fibroblasts with low migration and proliferation rates and lower production of elastin, fibrillin, and fibronectin observed in primary cultures.^1^

When the skin is artificially stretched during surgical stretching procedures using skin expanders, an incredibly large area of skin is obtained. The epidermis is stretched, and the skin thickens in response to the stretching.^2^ The tonofibrils in the basal cell layer and stratum spinosum (prickle cell layer) have larger bundles of tonofilaments compared to normal skin. Increased mitotic activity of the epidermis during skin expansion has been demonstrated.^3,4^ This situation results in hyperactivity of the physiologic repair mechanism in the surgical scar created during skin expander positioning. Hypertrophic scars and keloids are widely recognized as mechanically induced fibroproliferative skin diseases.^5^

## Methods

The medical records of all patients were reviewed and retrospectively evaluated. A total of 14 patients who presented to Mexico City General Hospital, Mexico, between May and December 2020 were included in the current study. We included data from patients who underwent breast reconstruction after breast cancer. The exclusion criteria were the presence of comorbidities directly affecting the wound healing process, diabetes mellitus, and autoimmune diseases. Patients who met these criteria were invited to participate in the study, and no changes were carried out to their therapy. They gave consent for their histologic results to be used for study purposes only. Breast reconstruction with a latissimus dorsi flap and an expander was carried out in the operating room for all patients. As part of the hospital routine, a biopsy was obtained from the scar area of the previous surgery. The pathology team (without intervention or knowledge of the study) analyzed the biopsy results. Two weeks after the surgical procedure, a topical Cicolea cream® (Paris-France), a cosmeceutical complement, was prescribed and applied to the fully closed areas as part of general care, provided there were no bloody areas or active infectious processes. Patients followed the hospital protocol for tissue expansion for 8-16 weeks. They then returned to the operating room for the second reconstruction phase, during which the expander was removed and the breast implant was placed. During the tissue expansion phase (in the time between the first and second surgery), 7 patients used Cicolea cream at least twice a day. The remaining patients used petroleum jelly without taking any further measures. In the second operation, part of the surgical scar was treated with Vaseline or removed. In the latter case, the sample was sent to the Pathology Department for histopathological examination.

All procedures carried out in this study were in accordance with the 1964 Declaration of Helsinki and its subsequent amendments or comparable ethical standards, and the protocol was approved by the local ethics committee of Mexico City General Hospital, Mexico. Written informed consents for the use of patient information and images were obtained from a legally authorized representative of each patient.

### Statistical analysis

Demographic data were collected from each patient who participated in the study as part of hospital logistics, and a comparative mean analysis was carried out. Descriptive analysis was carried out using frequencies and percentages for categorical variables and means, standard deviations, medians, and interquartile ranges (IQR) for continuous variables.

## Results

**Figure 1:**
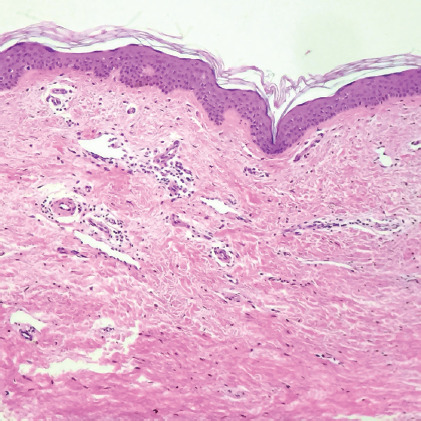
- Histology of a scar following treatment with Cicolea. The epidermis has recovered a normal thickness and a regular organization with epidermal crests separated by superficial dermal papillae. The deeper dermis contains organized collagen fibers, which are still relatively thick in comparison to a normal dermis but are not hyalinized (x10).

**Figure 2:**
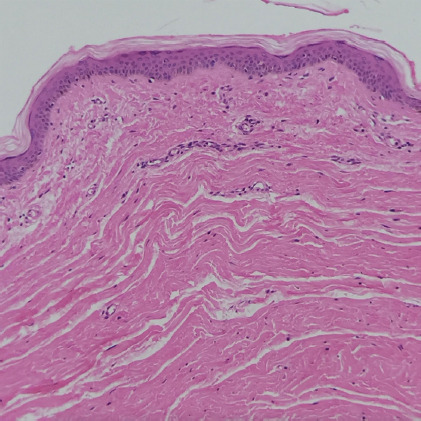
- Histology of a scar following petrolatum treatment. The epidermis is thin and relatively atrophic with a flattened basement membrane and an almost total absence of epidermal crests. The dermis, with no clear distinction between a superficial and a deep zone, contain collagen fibers which are both thick and hyalizined (H&E 40x).

The mean age of the 14 patients included in the current study was 51 years (range: 38-65). In terms of socioeconomic level, most patients belonged to the middle class. They had varying levels of education, ranging from elementary to undergraduate. The average number of years after cancer cure was 7 years. Approximately 90% of the patients received 20 cycles of radiotherapy and approximately 10 sessions of chemotherapy for cancer treatment. All patients underwent breast reconstruction with a latissimus dorsi flap with expansor. One patient had a late hematoma and required reoperation one month after the initial procedure. One patient experienced active bleeding 6 hours after the surgical procedure and required immediate reoperation for donor site hemostasis. A total of 7 patients were randomly selected for treatment with Cicolea cream® and the others received petrolatum. Patients were thus divided into 2 groups: those who used Cicolea cream® as a treatment adjunct and those who used petrolatum alone. All patients were taking antibiotics and non-steroidal anti-inflammatory drugs as analgesics, and none of them experienced infectious complications. We found histological differences between the group of patients treated with Cicolea-crem® and those treated with petrolatum. That is, regularly arranged collagen fibers were found in the group treated with Cicolea-Creme® (Figure 1), while scattered collagen fibers were found in the group treated with petrolatum (Figure 2). Different results were also observed in the patients with hypertrophic or keloid scars (Table 1), which had occurred after mastectomy or after the insertion of breast expanders. In these patients, the results of the histologic examination of the scar removed during the first procedure were compared with those of the histologic examination of the scar removed during the second procedure. The histological examination showed that the patients treated with Cicolea cream® had an orderly scar process with improvement of the scar. In contrast, most of the patients treated with petrolatum (57%) showed disorganized collagen patterns with hypertrophic scar recurrence.

**Table 1:** - Demographic data and histopathological results of the patients included in the current study.

Patient’s no.	Product	age	Localisation	Surgical procedure	Histology scar before procedure	Histology scar after procedure
1	cicolea	54	Scar on rigt breast	Latissmus dorsi flap expandend	fibrous scar with chronic inflammation	organized scar process
2	cicolea	47	Scar on rigt breast		fibrous scar with chronic inflammation	scar process
3	cicolea	61	Scar on rigt breast		fibrous scar	organized scar process
4	cicolea	64	bilateral breast scar		fibrous scar	organized scar process
5	cicolea	62	left breast scar		fibrous scar	scar process
6	cicolea	38	rigt breast scar		fibrous scar	scar process
7	cicolea	54	left breast scar		fibrous scar	scar process
8	Petrolatum	50	Scar on rigt breast		fibrous scar	scar process
9	Petrolatum	58	left breast scar		fibrous scar with granuloma process	scar process
10	Petrolatum	42	left breast scar		fibrous scar	Non organized scar process
11	Petrolatum	35	left breast scar		fibrous scar	Non organized scar process
12	Petrolatum	50	Scar on rigt breast		fibrous scar	scar process
13	Petrolatum	58	left breast scar		fibrous scar with cronic inflammation	Non organized scar process
14	Petrolatum	50	Scar on rigt breast		fibrous scar	Non organized scar process

## Discussion

The ideal scar is obtained in fetal surgery, but in adults other forces influence the physiological mechanisms of the scar. Normally, when the scar develops physiologically, it is considered immature until the end of the fourth week. The remodeling phase extends for 6-12 months after the original surgery or trauma. During this period, the ratio of type 1/3 collagen is 2/1 (33% type 3 collagen), but the collagen preserved at the end of the immature phase changes. Matrix metalloproteinase (MMP) activity destroys type-3 collagen and produces predominantly organized type-1 collagen, corresponding to skin tension lines. The ratio of type 1/3 collagen typically found in normal skin is 4/1.^6^ Ultimately, it is possible that mechanical tension forces influence all phases of wound healing. During skin expansion after implantation of the skin expander prosthesis, mechanical forces simultaneously stimulate keratinocytes and fibroblasts. Several studies have shown that mechanical stretching triggers antiapoptotic signals during skin expansion, as proliferation and antiapoptotic signals often occur simultaneously. Apoptosis is programmed: cell death is triggered by various stimuli such as interferon gamma, tumor necrosis factor alpha (TNF-α), and ultraviolet radiation. Mechanical stretch stimulates the calcium channel, which inhibits protein kinase B (AKT) phosphorylation, leading to alteration of normal cellular apoptosis on the skin and scar and secondary accumulation of collagen and extracellular matrix (ECM).^7^ This situation favors the development of hypertrophic and keloid scars. Protein kinase B is one of the kinases known to suppress apoptosis (thus protecting keratinocytes from it), inhibit differentiation, and increase survival.^8^ In the current study, epidermal cells subjected to cyclic axial stretch were found to migrate faster. The cytoskeleton, which is downstream of signals mediated by focal adhesion, is actively involved in the functional and morphological effects of elongation. This suggests that stretching weakens cell adhesion, thereby promoting an active cell migration response to stretching.^9^ During such active cell migration, apoptosis is reduced; therefore, it seems unlikely that reduced apoptosis contributes significantly to ECM accumulation, especially under conditions of increased MMP expression. Cyclic stretch significantly regulates mRNA expression of transforming growth factor beta 1 (TGF-β1). It is known to play an important role in mechanotransduction of scars, and acyclic axial stretching results in significant morphological and functional effects on fibroblasts.^10^ The stretched fibroblasts migrate faster, move farther, and realign perpendicular to the direction of stretching. This is accompanied by a decrease in cellular apoptosis but unchanged cellular proliferation. Furthermore, although stretch did not alter collagen synthesis, it did increase MMP expression. Matrix metalloproteinases are known to play a role in collagen degradation and possibly in increased fibroblast migration.^11^ Increased nitric oxide and TGF-β1 levels and lower TNF-α levels with concomitant blockade of apoptosis and MMP activity lead to excessive collagen and fibroblast accumulation. Based on our observations, it is possible to obtain altered wound healing with hypertrophic or keloid scar during skin expansion. However, in our clinical practice, we have observed that patients treated with Cicolea cream® during skin stretching have better scar quality than patients treated with petrolatum. Cicolea cream® is a natural formulation containing several plant extracts. These extracts act synergistically to help fibroblast and keratinocyte cells simultaneously physiologically repair skin after injury and prevent keloid scar formation.^12-14^ Petrolatum is a petroleum product commonly used in dermatology because it is non-irritating; it is an emollient and very safe for human skin. It is very well tolerated by the skin and has the same occlusive potential as vegetable oil.^15^ This product, often used in cosmetic formulations, is optimal for covering the skin after superficial injuries to protect it from bacterial contamination, but it does not directly contribute to cellular activity during wound healing. Most of the scars treated with Cicolea cream® developed into scars with better collagen fiber arrangement and a significant reduction or absence of collagen hyalinosis. We observed non-hypertrophic or keloid scars in patients treated with Cicolea cream® and compared them with those of patients treated with petrolatum.

### Study limitations

The main limitation of our study is the small sample size. In addition, although the data were collected prospectively, the analysis was retrospective and therefore subject to the inherent limitations of retrospective analyses.

In conclusion, we propose that the effect of polyphenols contained in the various ingredients of Cicolea Cream® results in better wound development or healing than the effect of petrolatum ingredients. In addition, the result of the scar treated with Cicolea cream® was also better when mechanical stretching was applied during skin stretching.
